# Electrochemical and AFM Characterization of G-Quadruplex Electrochemical Biosensors and Applications

**DOI:** 10.1155/2018/5307106

**Published:** 2018-01-31

**Authors:** Ana-Maria Chiorcea-Paquim, Ramon Eritja, Ana Maria Oliveira-Brett

**Affiliations:** ^1^Department of Chemistry, Faculty of Sciences and Technology, University of Coimbra, 3004-535 Coimbra, Portugal; ^2^CIBER-BBN, Institute for Advanced Chemistry of Catalonia (IQAC), Spanish Council for Scientific Research (CSIC), Jordi Girona 18-26, 08034 Barcelona, Spain

## Abstract

Guanine-rich DNA sequences are able to form G-quadruplexes, being involved in important biological processes and representing smart self-assembling nanomaterials that are increasingly used in DNA nanotechnology and biosensor technology. G-quadruplex electrochemical biosensors have received particular attention, since the electrochemical response is particularly sensitive to the DNA structural changes from single-stranded, double-stranded, or hairpin into a G-quadruplex configuration. Furthermore, the development of an increased number of G-quadruplex aptamers that combine the G-quadruplex stiffness and self-assembling versatility with the aptamer high specificity of binding to a variety of molecular targets allowed the construction of biosensors with increased selectivity and sensitivity. This review discusses the recent advances on the electrochemical characterization, design, and applications of G-quadruplex electrochemical biosensors in the evaluation of metal ions, G-quadruplex ligands, and other small organic molecules, proteins, and cells. The electrochemical and atomic force microscopy characterization of G-quadruplexes is presented. The incubation time and cations concentration dependence in controlling the G-quadruplex folding, stability, and nanostructures formation at carbon electrodes are discussed. Different G-quadruplex electrochemical biosensors design strategies, based on the DNA folding into a G-quadruplex, the use of G-quadruplex aptamers, or the use of hemin/G-quadruplex DNAzymes, are revisited.

## 1. Introduction

**Scheme 1:**
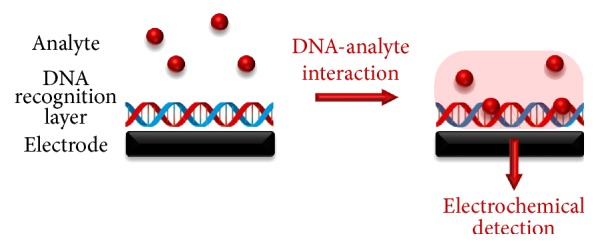
DNA-electrochemical biosensor: the analyte interaction with the DNA recognition layer immobilized at the electrode surface is electrochemically detected.

In addition to its genetic role, DNA represents one of the most important and smart self-assembling nanomaterials, being largely used in DNA nanotechnology and biosensor technology [1]. A DNA-electrochemical biosensor is a sensing device composed of a DNA layer (the biological recognition element) immobilized on the electrode surface (the electrochemical transducer), to detect target analytes that interact with DNA at nanoscale. The analytes will induce morphological, structural, and electrochemical changes in the DNA layer, which are further translated into an electrochemical signal, Scheme 1 [2–9]. The DNA-electrochemical biosensors are very robust, easy to miniaturise, present excellent detection limits, use small analyte volumes, and have the ability to be used in turbid biofluids, which make them exceptional tools for rapid and simple on-field detection. They also represent good models for simulating nucleic acid interactions with cell membranes, specific DNA sequences, proteins, pharmaceutical drugs, and hazard compounds [2–11].

**Scheme 2:**
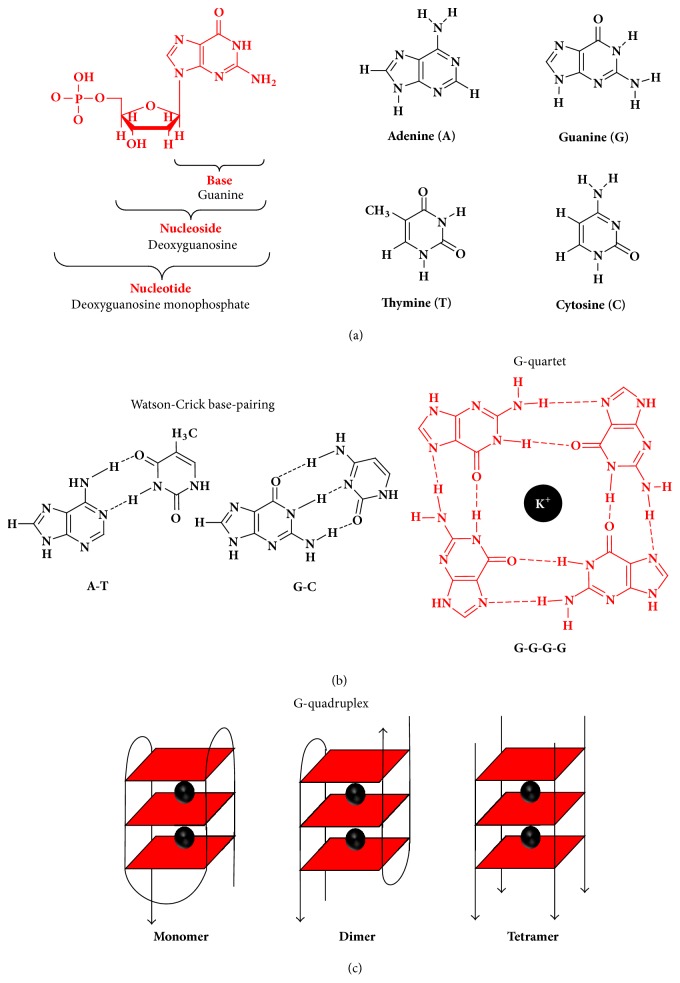
(a) Chemical structure of DNA nucleotides, nucleosides, and bases, (b) Watson-Crick base-pairing and G-quartet, and (c) G-quadruplex configurations. [Adapted from [11, 13] with permission.]

The DNA is composed of nucleotides, each containing a phosphate group, a sugar group, a nitrogen base, the purines adenine (A) and guanine (G), and the pyrimidines thymine (T) and cytosine (C), Scheme 2(a). The main structural conformation for natural DNA is the double-stranded DNA in Watson-Crick base pairs, Scheme 2(b), the cellular DNA being almost exclusively in this form [12]. However, DNA can be found in a variety of other conformations, such as double-helixes with different types of loops (bulge, internal, hairpin, junction, knotted loops, etc.), single-strands, triplex-helixes, or four-stranded secondary structures (e.g.,* i*-motifs and G-quadruplexes (GQs)) [11–13].

The GQs are four-stranded secondary structures, Scheme 2(c), formed by planar associations of four G bases, named G-quartets, held together by eight Hoogsteen hydrogen bonds, Scheme 2(b). The G-quartets are stacked on top of each other and stabilized by *π*-*π* hydrophobic interactions. Monovalent cations, such as K^+^ and Na^+^, are coordinated to the lone pairs of electrons of O^6^ in each G.

The GQ structures are polymorphic, and a variety of topologies have been observed by nuclear magnetic resonance (NMR) or crystallography, either as native structures or complexed with small molecules [14–17].

According to the number of strands, GQs can be classified as monomers (unimolecular, intramolecular, e.g., the human telomeric DNA d[AG_3_(T_2_AG_3_)_3_] in the presence of K^+^ ions, Protein Data Bank (PDB) entry 1KF1 [18]), dimers (bimolecular, intermolecular, e.g., the* Oxytricha nova* telomeric sequence d(G_4_T_4_G_4_) in the presence of K^+^ ions, PDB entry 1JPQ [19]), or tetramers (tetramolecular, intermolecular, e.g., the* Tetrahymena *telomeric sequence d(TG_4_T) in the presence of Na^+^ and Ca^2+^ ions, PDB entry 2GW0 [20]), Scheme 2(c).

According to the strand polarity (i.e., the relative arrangement of adjacent strands), the GQs present parallel or antiparallel orientations, according to the glycosidic torsion angle, they present* anti* or* syn* orientation, and according to the orientation of the connecting loops, they can be lateral, diagonal, or both [21–24].

The GQ sequences are found in chromosomes' telomeric regions, oncogene promoter sequences, RNA 5′-untranslated regions (5′-UTR), and other relevant genome regions, where they may influence the gene metabolism process and also participate in DNA replication, transcriptional regulation, and genome stability [14, 21–32]. The GQ formation has been associated with a number of diseases, such as cancer, HIV, diabetes, and aging [14, 23]. They are also considered important cancer-specific molecular targets for anticancer drugs, since the GQ stabilization by small organic molecules can lead to telomerase inhibition and telomere dysfunction in cancer cells [22, 33, 34].

Due to GQs biological role, extraordinary stiffness, and the ability to self-organize in more complex two-dimensional networks and long nanowires, they have become relevant in structural biology, medicinal chemistry, supramolecular chemistry, nanotechnology, and biosensor technology [14, 22, 23, 25, 35–37].

Short chain G-rich DNA sequences that form GQ structures are now used as recognition elements in GQ electrochemical biosensor devices, since the electrochemical response is particularly sensitive to the DNA sequence structural variations from a single-stranded, double-stranded, or hairpin configuration into a GQ configuration. In addition, short aptamers able to form GQs received a great deal of attention, since they are highly specific in binding to small molecules, proteins, nucleic acids, and even cells and tissues. These GQ aptamers combine the G-quadruplex stiffness and self-assembling versatility with the aptamer high specificity of binding, which allowed the construction of GQ electrochemical biosensors with increased selectivity and sensitivity.

The recent advances on the characterization of the G-rich DNA sequences, at the surface of electrochemical transducers, and the design and applications of GQ electrochemical biosensors for the detection of metal ions, GQ ligands, and other small organic molecules, proteins, and cells will be presented.

## 2. Electrochemical and AFM Characterization of G-Quadruplexes

**Figure 1:**
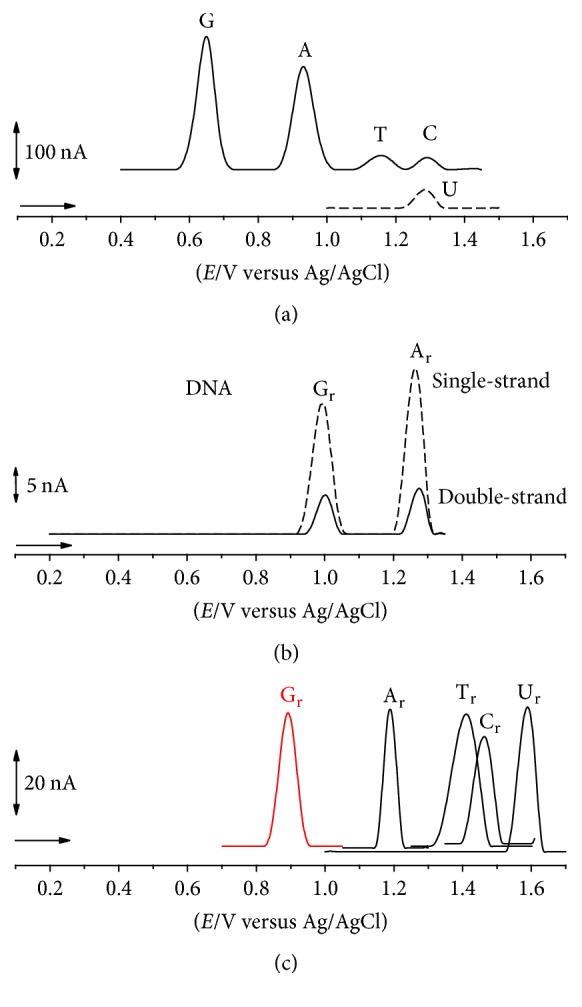
DP voltammograms baseline corrected at a GC electrode, solutions of ((a) black solid line) 20 *μ*M guanine (G), adenine (A), thymine (T), and cytosine (C); ((a) black dashed line) 20 *μ*M uracil (U) in pH 7.4; (b) 60 *μ*g mL^−1^ DNA (black solid line) double-strand and (black dashed line) single-strand in pH 4.5; ((c) red solid line) 40 *μ*g mL^−1^ poly(dG); ((c) black solid line) 40 *μ*g mL^−1^ poly(dA), 100 *μ*g mL^−1^ poly(dT), 100 *μ*g mL^−1^ poly(dC), and 250 *μ*g mL^−1^ poly(dU) in pH 7.4. [Adapted from [48, 49] with permission.]

Understanding the redox behaviour and adsorption process of the DNA probe at electrochemical transducers is critical for the design and successful application of DNA-electrochemical biosensors [2–7, 9–11, 38–46]. At carbon electrodes, the voltammetric studies showed that DNA bases, Figure 1(a), nucleosides, and nucleotides are all electroactive [47–49].

The double- and single-stranded DNA oxidation in solution shows two anodic peaks, corresponding to the G residues oxidation (G_r_) and A residues oxidation (A_r_), Figure 1(b) [2–7, 9–11, 38–46]. The T and C residues oxidation is more difficult to detect, since it occurs with a very low current, at very high positive potentials, near the potential of oxygen evolution.

The electrochemical behaviour of G-rich DNA sequences, able to self-assemble into GQ configurations, Scheme 2(c), was recently studied [54–59].

### 2.1. Short Chain G-Rich Oligonucleotides

The first report on the electrochemical oxidation of GQs concerned the investigation of two different length thrombin-binding aptamer (TBA) sequences, d(G_2_T_2_G_2_TGTG_2_T_2_G_2_) and d(G_3_T_2_G_3_TGT_3_T_2_G_3_) [54]. Both TBA sequences form chair-like unimolecular GQs in K^+^ ion containing solutions, consisting of two G-quartets connected by two TT loops and a single TGT loop. The different adsorption patterns and degree of surface coverage observed by atomic force microscopy (AFM) were correlated with the sequence base composition, presence/absence of K^+^ ions, and voltammetric behaviour observed by differential pulse (DP) voltammetry.

In the absence of K^+^, in Na^+^ containing solutions, the formation of GQs was very slow. The oxidation of both TBA sequences showed only one anodic peak, corresponding to the G residues oxidation in the TBA single-strands [54]. In the presence of K^+^, both TBA sequences folded into GQs. Since no adsorption of the stable and rigid GQs occurred, only a few single-stranded sequences were observed on the highly oriented pyrolytic graphite (HOPG) surface. DP voltammetry showed the decrease of the G residues oxidation peak and the occurrence of a new GQ peak at a higher potential, corresponding to the G residues oxidation in the GQs. The GQ higher oxidation potential was due to the greater difficulty of electron transfer from the inside of the GQ structure to the electrode surface compared to the electron transfer from the G residues in the more flexible single-strands.

**Figure 2:**
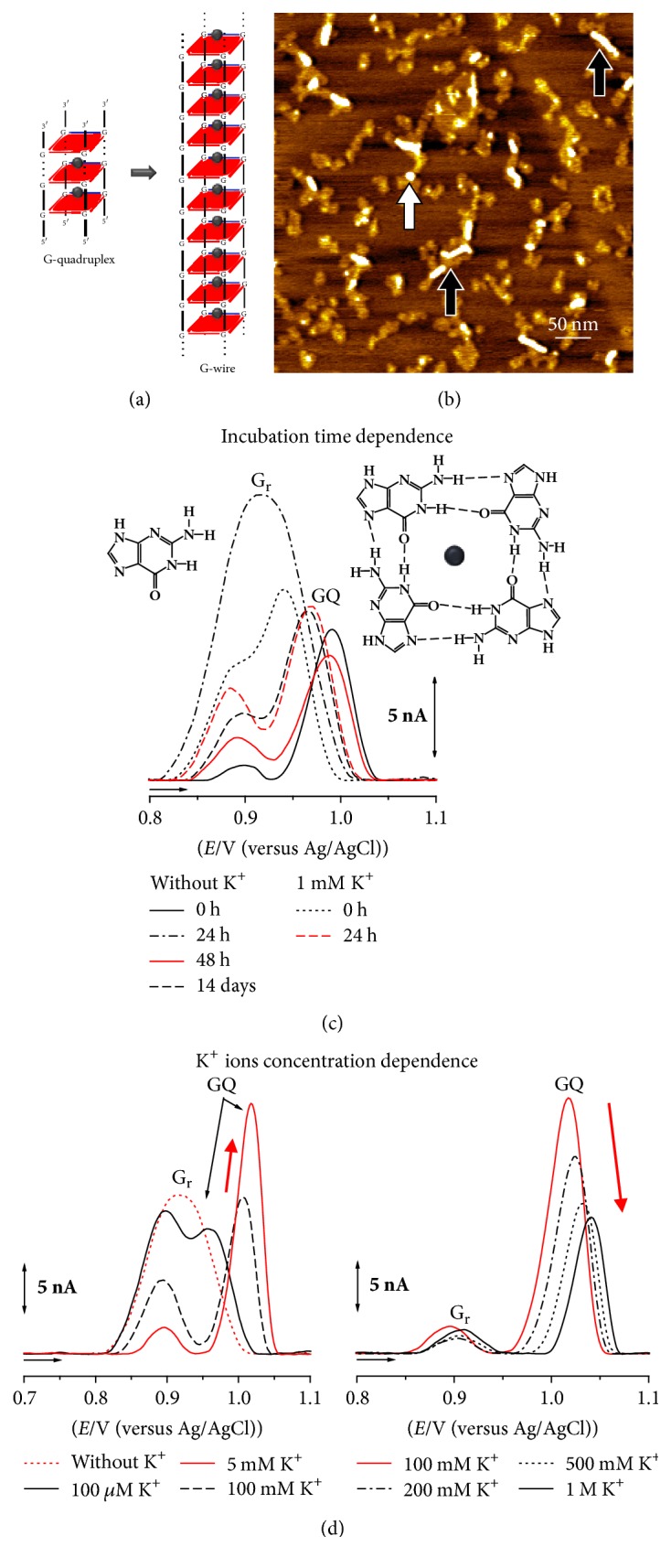
(a) Representation of d(G)_10_-GQ and GQ-based nanowire; (b) AFM image of 0.3 *μ*M d(G)_10_ in pH 7.0, 100 mM K^+^ ions, at 24 h incubation; (c, d) DP voltammograms baseline corrected in 3.0 *μ*M d(G)_10_ in pH 7.0: (c) incubation time dependence and (d) K^+^ ions concentration dependence at 0 h incubation. [Adapted from [50] with permission.]

The redox behaviour and adsorption of the d(G)_10_, d(TG_9_), and d(TG_8_T) oligodeoxynucleotide (ODN) sequences were studied by AFM and DP voltammetry at carbon electrodes [50, 56, 57]. All sequences fold into parallel tetramolecular GQ structures, Scheme 2(c)-right. The results demonstrated that GQ formation was directly influenced by the ODN sequence and concentration, pH, and presence of monovalent cations, Na^+^ versus K^+^, Figures 2(c) and 2(d). DP voltammetry allowed the detection of the ODN single-strands folding into GQs and G-based nanostructures, Figure 2(a), in freshly prepared solutions, at concentrations 10 times lower than usually detected using other techniques generally employed to study the GQ formation.

Single-stranded d(G)_10_, d(TG_9_), and d(TG_8_T) ODNs, in Na^+^ containing solutions and for short incubation times, were detected using AFM as network films and polymeric structures, Figure 2(b), and using DP voltammetry by the occurrence of only the G residues oxidation peak (G_r_), Figure 2(c) [13, 50].

GQ structures, in Na^+^ containing solutions and for long incubation times, or in K^+^ containing solutions, were detected using AFM as spherical aggregates, Figure 2(b) (white arrows). DP voltammetry showed the decrease of the G residues oxidation peak and the GQ oxidation peak occurrence, increase, and shift to more positive potentials, in a time-dependent manner, Figure 2(c) [13, 50]. Concerning the self-assembling into higher-order nanostructures, the homo-ODN sequence d(G)_10_ was the only sequence forming G-nanowires observed using AFM, Figure 2(b) (black arrows), d(TG_9_) formed short G-based superstructures that were adsorbed as rod-like shape aggregates, and d(TG_8_T) formed no nanostructures, due to the presence of T residues at both 5′ and 3′ ends [50].

**Figure 3:**
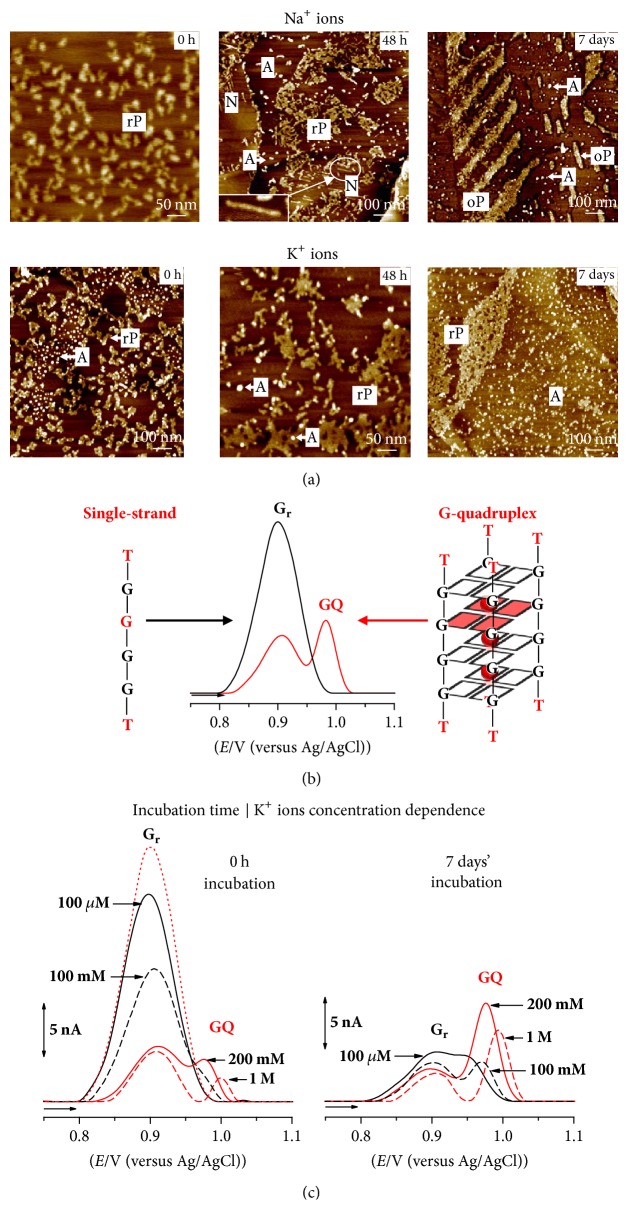
(a) AFM image of 0.3 *μ*M d(TG_4_T) in pH 7.0, in the presence of Na^+^ and K^+^ ions, at different incubation times; (b) representation of the d(TG_4_T) single-strand and GQ electrochemical detection; (c) DP voltammograms baseline corrected in 3.0 *μ*M d(TG^4^T) in pH 7.0: incubation time and K^+^ ions concentration dependence. [Adapted from [51] with permission.]

The d(TG_4_T) telomeric repeat sequence of the free-living ciliate protozoa* Tetrahymena* forms tetramolecular GQs that are considered simpler models of biologically relevant human quadruplexes, being used to obtain high resolution data on pharmaceutical drug-DNA interactions [51]. The well-known conformation of the d(TG_4_T) GQ and its extraordinary stiffness enabled the d(TG_4_T) to be considered a good building block candidate for the development of novel devices, with medical and nanotechnology applications. The d(TG_4_T) self-assembling from single-strand into GQ, influenced by the Na^+^ versus K^+^ ions concentration, was successfully detected using AFM on HOPG, Figure 3(a),  and DP voltammetry at glassy carbon (GC) electrode, Figures 3(b) and 3(c) [51]. The d(TG_4_T) GQs self-assembled very fast in K^+^ and slowly in Na^+^ containing solutions, revealing a time and a K^+^ ions concentration dependent adsorption process and redox behaviour, Figure 3(c).

AFM images of d(TG_4_T) spontaneously adsorbed from freshly prepared solutions (0 h incubation), in the presence of Na^+^ ions, showed only randomly oriented polymeric structures of 0.89 ± 0.1 nm height, due to the adsorption of single-stranded d(TG_4_T) molecules, as shown in the high amplification image from Figure 3(a)-up-left.

AFM images after 48 h incubation showed three adsorption morphologies: randomly oriented polymeric structures and network films of 0.81 ± 0.1 nm height, due the adsorption of d(TG_4_T) single-strands, spherical aggregates of 2.15 ± 0.6 nm height, due to the adsorption of short tetramolecular d(TG_4_T) GQs, and sporadically short nanowires of 0.80 ± 0.1 nm height and length up to 100 nm. In order to be able to distinguish the presence of the three morphologies, a larger amplification image, Figure 3(a)-up-middle, has been chosen.

AFM images after 7 days' incubation also showed three adsorption morphologies: very rarely, randomly oriented polymeric structures and network films of 0.81 ± 0.1 nm height, due the adsorption of d(TG_4_T) single-strands, spherical aggregates of 2.05 ± 0.5 nm height, due to the adsorption of d(TG_4_T) GQs, and oriented polymeric domains of 0.81 ± 0.1 nm height, adsorbed along one of the three axes of symmetry of the HOPG basal planes. Again, in order to distinguish the three adsorption morphologies, a larger amplification image, Figure 3(a)-up-right, has been presented.

AFM images of d(TG_4_T) immediately after the addition of K^+^ ions (0 h incubation) showed two adsorption morphologies: randomly oriented polymeric structures of 0.71 ± 0.2 nm height, due the adsorption of d(TG_4_T) single-strands, and spherical aggregates of 1.87 ± 0.4 nm height, due to the adsorption of d(TG_4_T) GQs, Figure 3(a)-down-left. Increasing the incubation time to 48 h (larger magnification image from Figure 3(a)-down-middle) and 7 days' incubation (Figure 3(a)-down-right), the number of 1.85 ± 0.5 nm height aggregates increased, while the 0.80 ± 0.1 nm height polymeric domains decreased.

The optimum K^+^ ions concentration for the formation of d(TG_4_T)-GQs was similar to the healthy cells intracellular K^+^ ions concentration. The d(TG_4_T) higher-order nanostructures self-assembled slowly in Na^+^ ion solutions and were detected by AFM as short nanowires and nanostructured films, Figure 3(a)-up. The absence of higher-order nanostructures in K^+^ ion solutions, Figure 3(a)-down, showed that the rapid formation of stable GQs induced by the K^+^ ions is relevant for the good function of cells.

In another report, the discrimination between double-stranded and GQ DNA was achieved at the gold electrode surface [60], based on the selective interaction between a [Ru(NH_3_)_6_]^3+^ redox label and DNA sequences able to form GQs with different folding type and numbers of G-quartets, d(G_2_T_2_G_2_TGTG_2_T_2_G_2_), d(T_6_G_2_T_2_G_2_TGTG_2_T_2_G_2_), d(AG_3_T_2_AG_3_T_2_AG_3_T_2_AG_3_), d(GTAG_2_TG_2_T_2_G_2_TGTG_2_T_2_G_2_), and d(T_6_CGTC_2_GTG_2_T2G_3_CAG_2_T_2_G_4_TGACT). A characteristic voltammetric peak was observed, due to a strong association between the [Ru(NH_3_)_6_]^3+^ redox label and the GQs, which was not detected for double-stranded DNA sequences.

### 2.2. Long Chain G-Rich Polynucleotides

Long chain polynucleotides poly(dG) and poly(G) are widely prevalent in the human and other genomes at both DNA and RNA levels and are used in DNA-electrochemical biosensors, as models to determine the preferential interaction of drugs with G-rich segments of DNA.

**Figure 4:**
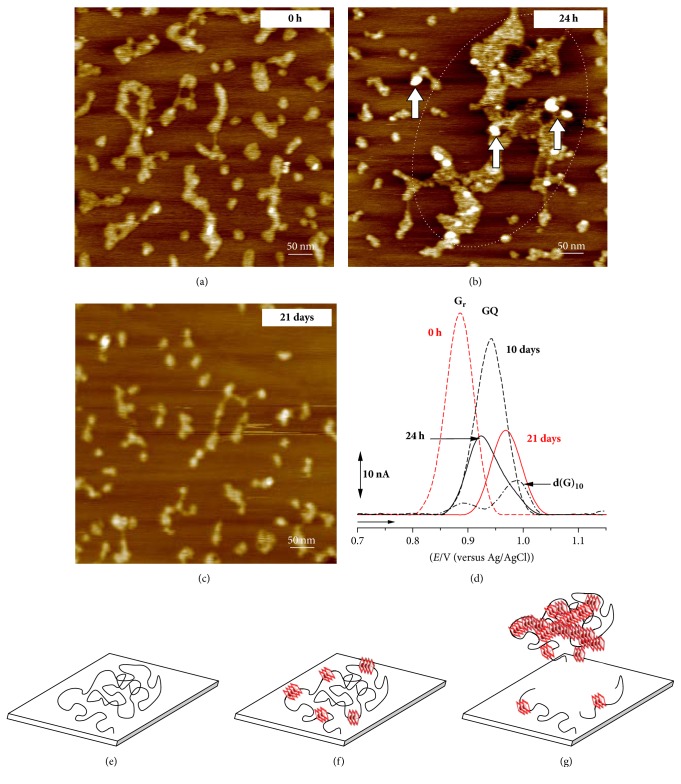
(a–c) AFM images of 5 *μ*g mL^−1^ poly(G) in pH 7.0, in the presence of K^+^ ions, at (a) 0 h, (b) 24 h, and (c) 21 days' incubation; (d) DP voltammograms baseline corrected in 100 *μ*g mL^−1^ poly(G) in pH 7.0, in the presence of K^+^ ions, at (red dashed line) 0 h, (black solid line) 24 h, (black dashed line) 10 days, and (red solid line) 21 days' incubation and control (black dashed-dotted line) 3 *μ*M d(G)_10_ at 24 h incubation; (e–g) representation of poly(G) adsorption process: (e) poly(G) single-strand, (f) poly(G) single-strand with short GQ regions, and (g) poly(G) single-strand with larger GQ regions. [Reproduced from [52] with permission.]

AFM at HOPG, Figures 4(a)–4(c), and DP voltammetric studies at GC electrode, Figure 4(d), showed that, in the presence of monovalent Na^+^ or K^+^ ions, the poly(G) single-strands self-assembled into short GQ regions for short incubation times. Large poly(G) GQ aggregates with low adsorption were formed after long incubation times, Figures 4(e)–4(g) [52]. DP voltammograms in freshly prepared poly(G) solutions showed only the G residues oxidation peak (G_r_), Figure 4(d), due to the G residues oxidation in the poly(G) single-strands.

Increasing the incubation time, the G residues oxidation peak decreased and disappeared, and a GQ oxidation peak in the poly(G) GQ morphology appeared, at a higher oxidation potential, dependent on the incubation time. The GQ oxidation peak current presented a maximum after 10 days' incubation and reached a steady value after ~17 days' incubation, Figure 4(d).

## 3. G-Quadruplex Electrochemical Biosensor Applications

Several electrochemical strategies are used in DNA-electrochemical biosensors applications:

- The direct label-free detection of DNA bases electrochemical current, monitoring the modifications of the G_r_ and A_r_ oxidation peaks
- The detection of redox reactions of reporter label molecules
- The detection of charge transport reactions mediated by the *π*-*π* interaction between DNA stacked bases.

However, for the design of GQ electrochemical biosensors, the use of redox molecular labels for electrochemical current amplification has been preferred.

The GQ electrochemical biosensor applications, for the detection of metal ions, GQ ligands and other small organic molecules, proteins, and cells, will be discussed.

### 3.1. Metal Ions

#### 3.1.1. Folding-Based G-Quadruplex Electrochemical Biosensor

**Scheme 3:**
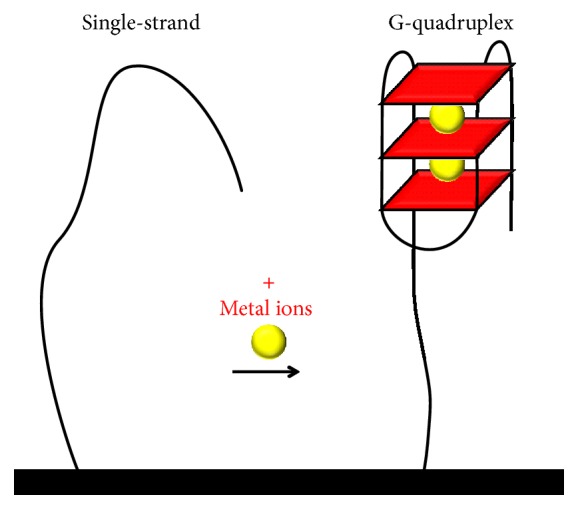
Folding-based GQ electrochemical biosensor for the detection of metal ions.

Besides the G-rich DNA sequence requirements, the coordination of monovalent cations is essential for the GQ formation and stability [61]. The physiologically relevant cations for GQ formation are the K^+^ and Na^+^, but, under specific conditions, cations such as Rb^+^, Cs^+^, NH_4_^+^, Tl^+^, Sr^2+^, Ba^2+^, and Pb^2+^ also influence the GQ formation. Based on the G-rich DNA conformational change from a single-strand to a GQ in the presence of metal ions, Scheme 3, different folding-based GQ electrochemical biosensors have been reported [62, 63].

A GQ electrochemical biosensor for K^+^ ions, based on the d(G_2_T_2_G_2_TGTG_2_T_2_G_2_) sequence switching from single-strand into a GQ in the presence of K^+^, was developed [64]. The ODN structural modifications were detected via changes on the electron transfer between a ferrocene (Fc) redox label and the gold electrode surface. Similar approaches were used for d(T_4_G_3_T_2_AG_3_T_2_AG_3_T_2_AG_3_) [65], d(T_22_CACATAGTCGACTCAG_3_) [66], and d(T_3_G_2_T_2_G_2_TGTG_2_T_2_G_2_T_3_) [67], self-assembled layers in the presence of Fc, either by voltammetry or electrochemical impedance spectroscopy (EIS). Folding-based GQ electrochemical biosensors for monitoring the Tb^3+^ ions at d[T_20_G_3_(T_2_AG_3_)_3_] [68] and Pb^2+^ at d(T_5_C_2_A_2_CG_2_T_2_G_2_TGTG_2_T_2_G_2_) [69] and modified gold electrodes, in the presence of Fc redox labels, were also described.

Small electroactive GQ ligands, able to intercalate into the GQ structure, such as ethyl green [62] and crystal violet [63], were also successfully employed as redox labels. Ethyl green GQ intercalator was applied for the development of a GQ electrochemical biosensor for the determination of Pb^2+^ ions [62], at the surfaces of carbon paste and multiwalled carbon nanotube paste electrodes. The electrochemical determination of Pb^2+^ was achieved by following the structural modification of the d(G_3_T)_4_ sequence from a single-strand into a GQ in the presence of Pb^2+^, followed by the ethyl green intercalation into the GQ, which caused changes in the ethyl green reduction peak current. In another example, a GQ electrochemical biosensor for Pb^2+^ detection was based on the electrochemical current of the crystal violet GQ ligand, with the d(G_3_T)_4_ sequence immobilized at a gold electrode surface [63].

#### 3.1.2. Hemin/GQ DNAzyme Electrochemical Biosensor

**Scheme 4:**
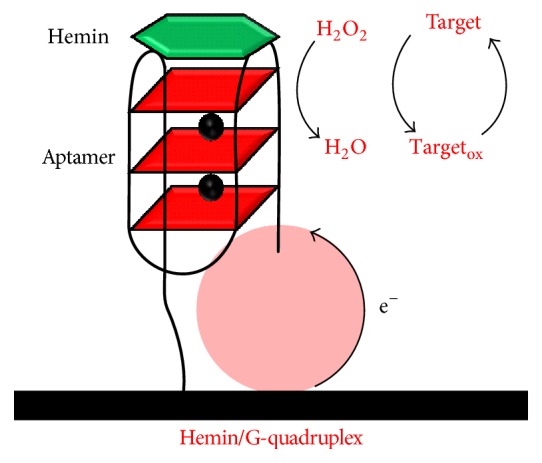
Hemin/G-quadruplex peroxidase-mimicking DNAzyme operating mode.

Hemin/GQ DNAzyme electrochemical biosensors represent one of the most popular building assays of GQ electrochemical biosensors [70]. In peroxidase hemin/GQ DNAzyme, the complex formed by hemin, an iron-containing porphyrin, with GQ DNA sequences, leads to an improved peroxidase activity of hemin and facilitates a redox reaction between a target molecule (the substrate, e.g., 3,3′,5,5′-tetramethylbenzidine, hydroquinone, or ferrocene methyl alcohol) and H_2_O_2_. The target molecule oxidation product is electrochemically detected, Scheme 4.

A hemin/GQ DNAzyme electrochemical biosensor for the detection of Hg^2+^ was developed [71], based on a bifunctional ODN sequence that contained a Hg-specific domain and a GQ domain, immobilized on gold electrode surface. The interaction between the GQ domain and hemin generated a hemin/GQ complex, which catalyzed the electrochemical reduction of H_2_O_2_, producing amplified readout currents for Hg^2+^ interaction events.

In a more complex design, the hemin/GQ DNAzyme was used to develop a surface plasmon resonance and electrochemical biosensor for Pb^2+^ ions [72]. A complex consisting of the Pb^2+^-dependent DNAzyme sequence and a ribonuclease-containing nucleic acid sequence (corresponding to the substrate of the DNAzyme) linked to a G-rich ODN sequence was assembled on gold electrode surfaces. In the presence of Pb^2+^ ions, the Pb^2+^-dependent DNAzyme cleaved the substrate, leading to the separation of the complex and to the self-assembly of the hemin/GQ complex. The electrochemical detection of Pb^2+^ showed a detection limit of 1 pM and a good selectivity.

In a different approach, taking advantage of the hemin/GQ ability to act both as a NADH oxidase, assisting the oxidation of NADH to NAD^+^ together with the generation of H_2_O_2_ in the presence of dissolved O_2_, and a peroxidase DNAzyme to bioelectrocatalyse the reduction of the produced H_2_O_2_, a hemin/GQ DNAzyme electrochemical biosensor for Hg^2+^ detection was developed [73]. The sensor showed improved detection limit and excellent selectivity against other interfering metal ions.

### 3.2. G-Quadruplex Ligands and Other Small Organic Compounds

#### 3.2.1. Folding-Based G-Quadruplex Electrochemical Biosensor

The GQ ligands are small molecules that bind to G-rich DNA sequences, which are considered novel therapeutic targets for anticancer drug development. They have the ability to induce and stabilize the DNA folding into GQ configurations at the level of telomeres, preventing the telomeric DNA from unwinding and opening to telomerase and thus indirectly targeting the telomerase and inhibiting its catalytic activity, which further leads to the senescence and apoptosis of tumour cells.

Recently, remarkable progress has been made in the development of selective GQ ligands that entered in clinical trials for cancer therapy, presenting significant telomerase inhibition or suppression of the transcription activity of oncogenes. Examples may include the trisubstituted acridine compound BRACO-19 and, more recently, a series of new triazole-linked acridine ligands, for example, GL15 and GL7, with enhanced selectivity for human telomeric GQ binding versus duplex DNA binding.

BRACO-19, GL15, and GL7 present complex, pH-dependent, and adsorption-controlled irreversible oxidation mechanisms, at GC electrode [74, 75]. The interaction between DNA and the acridine ligands GL15 and GL7 was investigated in incubated solutions and using DNA-, poly(G)-, and poly(A)-electrochemical biosensors [75]. Both GL15 and GL7 interacted with DNA in a time-dependent manner, with preferential affinity for the G-rich segments, but did not cause DNA oxidative damage.

**Figure 5:**
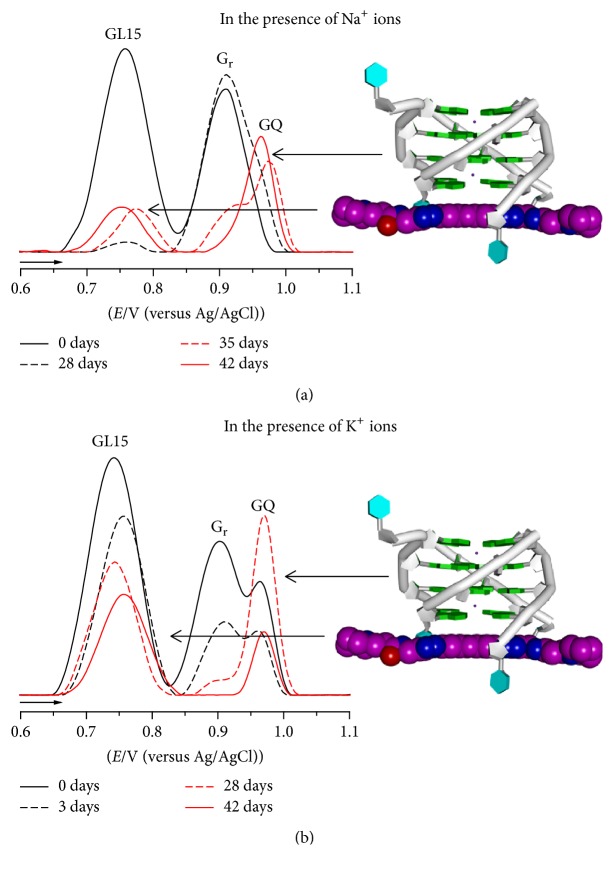
DP voltammograms baseline corrected in 3.0 *μ*M d(TG_4_T) incubated with 4.0 *μ*M GL15 in pH 7.0, at different incubation times, in the presence: (a) Na^+^ and (b) K^+^ ions. [Adapted from [53] with permission.]

The interactions of the GQ-targeting triazole-linked acridine ligand GL15 with the short chain length* Tetrahymena* telomeric DNA repeat sequence d(TG_4_T) and with the long poly(G) sequence have been studied [53]. The results showed that GL15 interacts with both sequences in a time-dependent manner. In the presence of GL15, GQ formation was detected by AFM via the adsorption of GL15-d(TG_4_T) GQ and GL15-poly(G) GQ small spherical aggregates and large GL15-poly(G) GQ assemblies and by DP voltammetry via GL15 and G_r_ oxidation peak current decrease and disappearance and the occurrence of a GQ oxidation peak, Figure 5.

The GL15 interaction with d(TG_4_T) and poly(G) was directly influenced by the presence in solution of monovalent Na^+^ ions, Figure 5(a), or K^+^ ions, Figure 5(b). These results were consistent with the interaction of triazole-linked acridine derivatives with terminal G-quartets in individual GQs, Figure 5-right. The binding, in Na^+^ or K^+^ ions solutions, of GL15 to d(TG_4_T) and poly(G) strongly stabilized the GQs and accelerated GQ formation, although only the K^+^ ions containing solution promoted the formation of perfectly aligned tetramolecular GQs [53].

An electrochemical biosensor for the investigation of GQ ligands telomerase inhibitors prepared by modification of a GC electrode with gold nanoparticles and GQ d((G_3_T_2_A)_4_G_3_) and* i-motif* d((C_3_TA_2_)_4_C_3_) DNA sequences was developed [76]. EIS results showed the increase of the charge transfer resistance with increasing the GQ ligand concentration, due to the GQ ligand interaction. 1,4-Dihydropyridine derivatives showed good GQ affinity in the concentration range from 5.0 to 700 *μ*M, and the ligands selectivity was studied using different control double-stranded DNA sequences.

A GQ electrochemical biosensor for the detection of GQ ligands ethidium bromide and polyamines spermine or spermidine was developed [77], which was based on the immobilization of the 30-mer d(G_3_(AG_3_)_2_A_2_G_2_(AG_3_)_3_AGC) sequence on a pretreated multiwalled carbon nanotubes modified GC electrode. The characteristics of the modified electrode and the GQ interaction with ethidium bromide and polyamines spermine or spermidine were investigated, via the [Ru(NH_3_)_6_]^3+^ peak current decrease with increasing ligands concentration.

Apart for GQ ligands, GQ electrochemical biosensors for the detection of other small organic molecules were developed, using aptamers with specific recognition for the target analyte. Several GQ aptamers for small organic molecules have been described in the literature, for example, hematoporphyrin IX, hemin, ochratoxin, and ATP [78], and some were already used for the development of GQ electrochemical biosensors.

Ochratoxin A (OTA) is a secondary metabolite of fungi strands like* Aspergillus ochraceus*,* Aspergillus carbonarius*, and* Penicillium verrucosum* that can contaminate a large number of food supplies. An impedimetric GQ electrochemical biosensor for the detection of OTA was developed, based on the OTA specific aptamer sequence d(GATCG_3_TGTG_3_TG_2_CGTA_3_G_3_AGCATCG_2_ACA) [79]. The aptamer was covalently immobilized onto a mixed Langmuir–Blodgett monolayer composed of polyaniline-stearic acid and deposited on indium tin oxide coated glass plates. The sensor showed a 0.24 nM detection limit for OTA [79]. This methodology was improved, a detection limit of 0.12 nM for OTA was achieved, and the biosensor has been also successfully applied for OTA determination in food samples [80].

Another design for the OTA detection proposed the use of a long polyethylene glycol spacer chain, which led to the formation of long tunnels at the surface of screen printed carbon electrodes, with the OTA aptamers acting as gates. The OTA specific binding to the aptamer led to changes in the aptamer configuration and, consequently, to a peak current decrease [81].

In a different approach, OTA was detected at a GQ electrochemical biosensor that used a hairpin OTA aptamer and site-specific DNA cleavage of the TaqaI restriction endonuclease and a streptavidin-horseradish peroxidase label [82].

#### 3.2.2. Hemin/GQ DNAzyme Electrochemical Biosensor

 A hemin/GQ DNAzyme electrochemical biosensor for the detection of the GQ ligands 5,10,15,20-tetra-(*N*-methyl-4-pyridyl) porphyrin (TMPyP4) and* N*,*N*-bis[2-(1-piperidino)-ethyl]-3,4,9,10-perylenetetracarboxylic diimide (PIPER) [83] was described. The biosensor was prepared using the human telomeric DNA sequence d(AG_3_(T_2_AG_3_)_3_) immobilized at the pyrolytic graphite electrode surface. Both the hemin and the GQ ligand bound simultaneously to the GQ structure, and neither PIPER nor TMPyP4 destroyed the hemin/GQ complex. Voltammetric and spectrometric methods were simultaneously employed to verify the interactions and binding stoichiometry between the GQ ligands and the hemin/GQ complex. The binding stoichiometry was determined to be 2 : 1 for TMPyP4-hemin/GQ and 4 : 1 for PIPER-hemin/GQ.

A common strategy for hemin/GQ DNAzyme electrochemical biosensors for the detection of small organic compounds that do not directly bind to the GQ consisted in the modification of the electrode surface by an ODN sequence that contains two domains. One domain is capable of forming a GQ structure, which binds the hemin and is used as amplification strategy. The other is an aptamer domain able to specifically bind the analyte, which may form or not a GQ structure. In the presence of the analyte and hemin, the hemin/GQ structures were formed on the electrode surface, while the analyte protein was bound to the aptamer part. This strategy was successfully used to design a hemin/GQ DNAzyme electrochemical biosensor for the detection of adenosine monophosphate (AMP) [84]. Upon interaction of a hairpin ODN sequence with AMP, the AMP-aptamer complex was formed, leading to the hairpin opening and the formation of the hemin/GQ complex. On the other hand, the adenosine deaminase was able to convert the AMP substrate into inosine monophosphate that lacked the affinity for the aptamer sequence. The sensor achieved a the 1 *μ*M detection limit for AMP [84].

Adenosine triphosphate (ATP) was detected at a hemin/GQ DNAzyme electrochemical biosensor that used two aptamers for ATP and for hemin recognition [85]. Two ODN sequences were designed, the first one immobilized on a gold electrode surface and containing the ATP aptamer and a part of the hemin aptamer and the second one containing the complementary strand of ATP aptamer and the rest of hemin aptamer. In the presence of ATP, the duplex between the two ODN sequences opened, the second ODN sequence diffused into the solution, and the hemin/GQ DNAzyme electrochemical current disappeared.

A dual-functional electrochemical biosensor for ATP and H_2_O_2_ from cancer cells was developed based on a hemin/G-quadruplex DNAzyme [86]. The double-stranded conformation of the ATP aptamer, immobilized on gold electrodes, changed upon ATP binding, forming a stable GQ, and a hemin/G-quadruplex DNAzyme, after addition of hemin, was formed. The electrochemical current of the Fc redox label increased in the presence of both ATP and H_2_O_2_.

A hemin/G4 DNAzyme based impedimetric biosensor was used to detect the environmental metabolite 2-hydroxyfluorene (2-HOFlu) [87]. Using the hemin/G4 peroxidase activity to catalyze the oxidation of 2-HOFlu by H_2_O_2_, the sensor achieved a 2-HOFlu detection limit of 1.2 nM in water and 3.6 nM in spiked lake water samples. The assay was also selective over other fluorene derivatives.

Hemin/GQ DNAzyme electrochemical biosensors for the detection of other organic compounds, such as the toxin microcystin-LR [88] and the pollutant agent naphthol [89], were also described.

### 3.3. Proteins

Due to aptamers' high selectivity, sensitivity, and reliability, the electrochemical biosensors are also very attractive tools for protein detection. TBA was the first aptamer observed in NMR studies to fold into a GQ structure [90], and it was shown that GQ formation is critical for thrombin specific recognition. TBA remains nowadays the most widely used aptamer in GQ electrochemical biosensors research. However, GQ aptamers that bind specifically to other protein targets have been selected, for example, nucleolin, signal transducer and activator of transcription STAT3, human RNase H1, protein tyrosine phosphatase Shp2, VEGF, HIV-1 integrase, HIV-1 reverse transcriptase, HIV-1 reverse transcriptase, HIV-1 nucleocapsid protein,* M. tuberculosis* polyphosphate kinase 2, sclerostin, and insulin [78].

#### 3.3.1. Folding-Based G-Quadruplex Electrochemical Biosensor

**Scheme 5:**
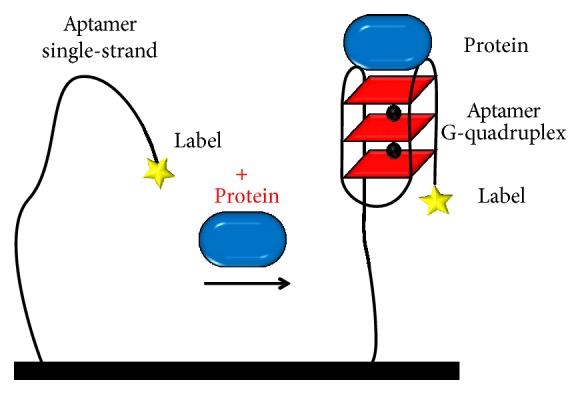
Folding-based GQ electrochemical biosensor for the detection of proteins.

Many GQ electrochemical biosensors for the detection of proteins are based on the aptamer structural modifications in the presence of the analyte, from a single-, double-strand, or hairpin configuration, into a GQ configuration. Generally, they consist in an aptamer modified with a redox label immobilized on the electrode surface, while the analyte is present in solution [91, 92], Scheme 5.

The first folding-based GQ electrochemical biosensor for thrombin was prepared by covalently attaching methylene blue (MB) labelled TBA sequences d(TA_2_GT_2_CATCTC_4_G_2_T_2_G_2_TGTG_2_T_2_G_2_T) [93] and d(T_2_C_2_A_2_CG_2_T_2_G_2_TGTG_2_T_2_G_2_) [94] to gold electrode surfaces. In the absence of thrombin, the immobilized TBA sequences remained unfolded, allowing the MB label to be in close proximity to the electrode surface and electron transfer occurred. Upon thrombin binding, the electron transfer between the MB redox label and the gold surface was stopped, due to the GQ aptamer formation. Similar GQ electrochemical biosensors for thrombin were developed, using the TBA sequence d(G_2_T_2_G_2_TGTG_2_T_2_G_2_) labelled with Fc [95–97], and Fe_3_O_4_-nanoparticles [98].

In another approach, the folding-based GQ electrochemical biosensor for thrombin was prepared by covalently attaching to a gold electrode an ODN sequence that contained a 15-base TBA sequence at its 3′ end and formed a double-helix with a MB-tagged partially complementary ODN sequence [99]. In the presence of thrombin, the 15-base TBA sequence self-assembled into a GQ, releasing the 5′ end of the MB-tagged ODN sequence as a flexible, single-stranded element and thus producing a detectable current. This strategy achieved an increase in current of ~300% with a saturated thrombin target, but the GQ electrochemical biosensor was not reusable. In a similar procedure, a TBA sequence d(C_2_A_2_CG_2_T_2_G_2_TGTG_2_T_2_G_2_) labelled with Fc immobilized on gold electrode was also employed [100].

A folding-based GQ electrochemical biosensor for thrombin was developed based on thrombin-induced split aptamer fragments conjunction [101]. The 15-base TBA sequence was split into two fragments, the d(A_6_G_2_T_2_G_2_TG) sequence that was attached to a gold electrode and the d(TG_2_T_2_G_2_T_6_) sequence modified with a Fc redox label. The thrombin-induced association of the two fragments increased the concentration of Fc at the gold surface, which was monitored by voltammetry.

A label-free impedimetric folding-based GQ electrochemical biosensor for thrombin was developed based on an electropolymerized poly(pyrrole-nitrilotriacetic acid) film onto the surface of a platinum electrode, followed by complexation of Cu^2+^ ions and immobilization of histidine-TBA sequences d(G_2_T_2_G_2_TGTG_2_T_2_G_2_T_5_) [102]. The biosensor presented high sensitivity for the detection and quantification of thrombin via EIS detection, without a labelling step.

Different electrode surface modification strategies have been used to improve the sensitivity of the GQ electrochemical biosensors, and examples may include the use of gold disk microelectrode arrays [103], gold electrode surface modified by polyamidoamine (PAMAM) dendrimer [104], GC electrode surface modified by gold nanoparticles [105] and multiwalled carbon nanotubes [106], magnetic nanobeads [107] and quantum dots-coated silica nanospheres [108], and gold nanoparticles [109] at graphite electrodes.

#### 3.3.2. Sandwich-Type GQ Electrochemical Biosensor

Many aptamers recognize specifically different positions on the analyte, as in the case of TBA that recognizes both the fibrinogen and heparin binding sites of thrombin, a property that was used for the development of sandwich-type GQ electrochemical biosensors.

**Scheme 6:**
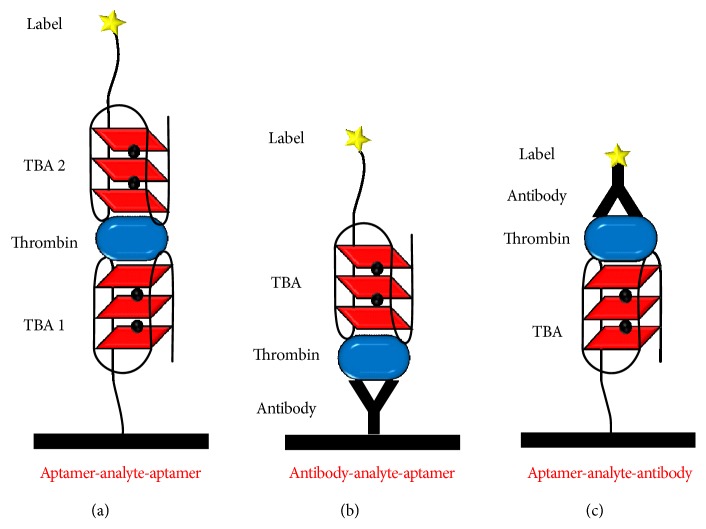
Sandwich-type GQ electrochemical biosensors for the detection of thrombin: (a) aptamer-analyte-aptamer, (b) antibody-analyte-aptamer, and (c) aptamer-analyte-antibody.

The first sandwich-type GQ electrochemical biosensor reported in the literature for thrombin detection presented an aptamer-analyte-aptamer format, Scheme 6(a), and led to the selective detection of 1 *μ*M of thrombin [110]. The sensor was built up by two aptamer layers, TBA 1 immobilized onto the gold electrode surface and used for capturing the thrombin analyte, and TBA 2, labelled with glucose dehydrogenase and used for the electrochemical detection, Scheme 6(a). The TBA sequences 15-mer d(G_2_T_2_G_2_TGTG_2_T_2_G_2_) and 29-mer d(AGTC_2_GTG_2_TAG_3_CAG_2_T_2_G_4_TGACT) were used as either TBA 1 or TBA 2, and similar results were obtained. In a very similar methodology [111], the 29-mer TBA was labelled with pyrroloquinoline quinone (PQQ) redox cofactor glucose dehydrogenase (GDH), which led to a current increase due to the electroactive product generated by the enzyme reaction and allowed the selective detection of more than 10 nM of thrombin.

The limit of detection of sandwich-type GQ electrochemical biosensor with aptamer-analyte-aptamer format was further lowered by employing different types of redox labels on the TBA 2, such as peroxidase [112], platinum nanoparticles [113], gold nanoparticles [114], and cadmium sulphide quantum dots [115, 116]. Using a more complex design, based on conductive graphene-3,4,9,10-perylenetetracarboxylic dianhydride nanocomposites as sensor platform, and PtCo nanochains-thionine-Pt-horseradish peroxidase labelled secondary TBA sequences d(G_2_T_2_G_2_TGTG_2_T_2_G_2_) for current amplification, a 6.5 × 10^−16^ M detection limit for thrombin was achieved [117].

A sandwich-type GQ electrochemical biosensor for thrombin detection with an antibody-analyte-aptamer format, Scheme 6(b), was also described [118]. The thrombin analyte was immobilized directly onto a nanogold-chitosan composite modified GC electrode surface, via a polyclone antibody. A 58-mer TBA sequence d(GACAGACGATGTGCTGACTACTG_2_T_2_G_2_TGAG_2_T_2_G_3_TAGTCAGCACATCGTCTGTC) labelled with MB was used for detection, and a 0.5 nM detection limit for thrombin was obtained [118].

In another approach, a sandwich-type GQ electrochemical biosensor for thrombin detection with an aptamer-analyte-antibody format, Scheme 6(c), was applied [119]. The sensor was built up by immobilizing the thrombin analyte to gold nanoparticles-doped conducting polymer nanorods via TBA d(G_2_T_2_G_2_TGTG_2_T_2_G_2_) sequences, the detection being performed with the Fc redox labelled antibody. The electrocatalytic oxidation of ascorbic acid by the Fc redox label allowed a low detection limit of 0.14 pM for thrombin. The biosensor was successfully tested in a real human serum sample for the detection of spiked concentrations of thrombin.

#### 3.3.3. GQ DNAzyme Electrochemical Biosensor

*(a) Hemin/GQ DNAzyme. *Based on the peroxidase hemin/GQs DNAzyme characteristics, Scheme 4, different hemin/GQ DNAzyme electrochemical biosensors were described. A hemin/GQ DNAzyme electrochemical biosensor, based on a TBA d(G_2_T_2_G_2_TGTG_2_T_2_G_2_) sequence, was applied for the thrombin detection [120]. In the presence of thrombin, the hemin/GQ DNAzyme activity increased, providing the amplified electrochemical readout currents, the sensor exhibiting high sensitivity and selectivity.

In a more complicated strategy, the thrombin detection was achieved using a dual current amplification scheme [121]. Gold nanoparticles were first electrodeposited onto single wall nanotube graphene modified electrode surface, for the immobilization of electrochemical probe of nickel hexacyanoferrates nanoparticles. Subsequently, another gold nanoparticles layer was electrodeposited for further immobilization of TBA sequences, which later formed the hemin/GQ DNAzyme. On the basis of the dual amplifying action, a detection limit of 2 pM for thrombin was obtained.

A hemin/GQ DNAzyme electrochemical biosensor for thrombin detection, based on background noise reduction by exonuclease I (Exo I), was also described [122]. The TBA sequences were self-assembled onto gold nanoparticles modified screen printed carbon electrode surfaces. In the absence of the target thrombin, the TBA sequences were digested by Exo I, which impeded the hemin association, significantly reducing the background current noise. The thrombin binding stabilized the TBA GQ and prevented it from being degraded by Exo I, the hemin/GQ complex formation generating the amplified electrochemical current. The introduction of Exo I significantly enhanced the current to noise ratio of the electrochemical sensor response.

An electrochemical aptasensor for thrombin that used the cocatalysis of hemin/GQ DNAzyme and octahedral Cu_2_O-Au nanocomposites for signal amplification was designed [123]. Gold nanoparticles were grown directly on the surface of the octahedral Cu_2_O nanocrystals. The Cu_2_O-Au nanocomposites obtained were simultaneously used for signal amplifying molecules and as nanocarriers. The hemin/GQ DNAzyme was formed by intercalating hemin into the d(G_2_T_2_G_2_TGTG_2_T_2_G_2_) TBA sequences and the electroactive toluidine blue and was immobilized onto the Cu_2_O-Au nanocomposite surfaces. The aptasensor exhibited a detection limit of 23 fM for thrombin, good sensitivity, and high specificity [123].

Based on the hemin/GQs that can simultaneously act as NADH oxidase and peroxidase DNAzyme, different hemin/GQ DNAzyme electrochemical biosensors for the detection of thrombin were reported [124–127].

A pseudo triple-enzyme cascade electrocatalytic electrochemical biosensor for the determination of thrombin used the amplification of alcohol dehydrogenase-Pt-Pd nanowires bionanocomposite and hemin/GQ structure that simultaneously acted as NADH oxidase and peroxidase DNAzyme [127]. The alcohol dehydrogenase immobilized on the Pt-Pd nanowires catalyzed the ethanol present in the electrolyte into acetaldehyde, accompanied by NAD^+^ being converted to NADH. Then the hemin/GQ acted first as NADH oxidase, converting the produced NADH to NAD^+^, and then the hemin/GQ complex acting as peroxidase DNAzyme catalyzed the reduction of the produced H_2_O_2_.

Another strategy used porous platinum nanotubes labelled with glucose dehydrogenase and hemin/GQ complexes acting as both NADH oxidase and peroxidase DNAzyme, which led to a cascade signal amplification and allowed the detection limit of thrombin down to 0.15 pM level [128].

The Pebrine disease related* Nosema bombycis* spore wall protein was detected at a hemin/GQ DNAzyme electrochemical biosensor [129], using the amplification of hemin/GQ DNAzyme functionalized with Pt-Pd nanowires, that again acted as both NADH oxidase and peroxidase, the sensor exhibiting good linear range and detection limit.

**Scheme 7:**
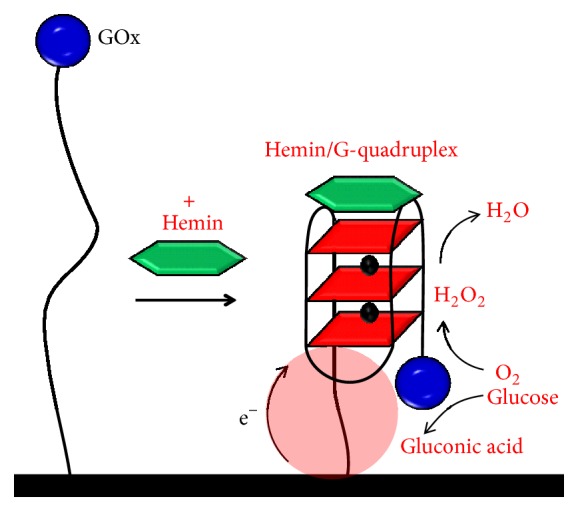
Hemin/G-quadruplex DNAzyme electrochemical biosensor for the detection of glucose.

Several reports on hemin/GQ DNAzyme electrochemical biosensors used the electrocatalytic properties of the DNAzyme to detect the activities of enzymes and their substrates. In fact, the first hemin/GQ DNAzyme electrochemical biosensor study followed the glucose oxidase (GOx) activity, by attaching the GOx to the gold electrode surface through a nucleic acid sequence able to form GQs in the presence of hemin, Scheme 7 [84]. The GOx mediated the glucose oxidation to gluconic acid and H_2_O_2_ and the H_2_O_2_ detection by its electrocatalyzed reduction by the DNAzyme. The concentration of H_2_O_2_ generated upon the interaction of the modified electrode with glucose, was proportional to the concentration of glucose, and the system could quantitatively determine the glucose substrate. In another report, a multiple current amplification strategy for glucose detection, based on hollow PtCo nanochains functionalized by DNAzyme and GOx, as well as Fc-labelled secondary TBA sequences [130], proved to be able to distinguish the target protein from interfering molecules.

Mammalian Argonaute 2 (Ago2) protein is the key player of RNA-induced silencing complexes, regulating gene function through RNA interference. A hemin/GQ DNAzyme electrochemical biosensor for the detection of Ago2 protein and study of its RNA endonuclease activity was developed [131]. A hairpin ODN structure, which contained a GQ domain that recognized the hemin and a domain that specifically recognized the target mRNA sequences complex with Ago2, was immobilized onto gold electrode surface. In the presence of Ago2, the hairpin was cleaved into two pieces and the hemin region become free and formed a stable hemin/GQ complex in the presence of K^+^ ions. The results showed that Ago2 catalyzed the cleavage of target RNA in the absence of any biological partners or ATP and maintained the catalytic activity, in a wide range of pH and temperature.

The adenosine deaminase (ADA) activity was detected at a hemin/GQ DNAzyme electrochemical biosensor [132], which employed an ODN sequence with three functional domains, an adenosine aptamer domain, a GQ domain, and a linker domain, immobilized on a gold electrode. In the presence of adenosine, the adenosine aptamer formed a close-packed tight structure with the adenosine. However, upon addition of ADA in the test solution, adenosine was converted into inosine due to the catalytic reaction, and the release of inosine made the adenosine aptamer region flexible again. The ADA inhibition was also studied in the presence of erythro-9-(2-hydroxy-3-nonyl) adenine hydrochloride inhibitor.

A DNA-based electrochemical method for the detection of alkaline phosphatase (AP) activity has been developed that used a DNA polymerase terminal deoxynucleotidyl transferase (TdT) and hemin/GQ DNAzyme nanowires acting as both NADH oxidase and peroxidase [133]. A 3′-phosphorylated double-stranded DNA probe was immobilized on a gold nanoparticles modified GC electrode. In the presence of AP, the 3′-phosphoryl end of the DNA probe becomes dephosphorylated, and the TdT catalyzed the DNA probe extension with a poly(T) sequence. Then, a G-rich DNA strand was hybridized with the poly(T) sequence of the DNA probe, which then formed a hemin/GQ DNAzyme in the presence of hemin. In the presence of NADH, the hemin/GQ DNAzyme oxidized NADH to NAD^+^, accompanied by the formation of H_2_O_2_, which was further catalyzed by the DNAzyme. The developed biosensor presented good sensitivity, selectivity, reproducibility, and stability, showing promising practical applications in AP activity assay.

*(b) Cu^2+^/GQ DNAzyme. *Besides hemin, it was demonstrated that the human telomeric DNA assembled with Cu^2+^ ions can present DNAzyme activity, being able to catalyze the Friedel-Crafts reaction in water with excellent enantioselectivity [134, 135]. Based on a Cu^2+^/GQ DNAzyme, an electrochemical method for pyrophosphatase (PPase) activity detection was developed [136]. In the absence of PPase, Cu^2+^ coordinated with pyrophosphate (PPi) to form a Cu^2+^-PPi compound. In the presence of PPase, the PPase catalyzed the hydrolysis of PPi into inorganic phosphate and produced free Cu^2+^, which self-assembled to the G-rich DNA on the screen printed gold electrode surface and formed a Cu^2+^/GQ DNAzyme. Using 3,3′,5,5′-tetramethylbenzidine as a redox mediator, the Cu^2+^/GQ DNAzyme catalyzed the reduction of H_2_O_2_ to generate a quantitative chronoamperometric signal. This method was additionally applied to screen the sodium fluoride inhibitor for PPase [136].

### 3.4. Cancer Cells

With the increase demand on the development of new strategy for cancer early detection, GQ electrochemical biosensors have the potential to be important tools for cancer cell detection in early cancer diagnosis. A hemin/GQ DNAzyme electrochemical biosensor for the detection of human liver hepatocellular carcinoma cells (HepG2) was proposed [137], based on the thiolated TLS11a aptamer attached to a gold electrode specific recognition of the target HepG2 cells. Hemin/GQ modified gold nanoparticles were also used for current amplification. After the electrochemical detection, the activation potential of −0.9 to −1.7 V was used to regenerate the gold electrode surface, the gold electrode showing good reusability.

In another approach, the HepG2 cells were detected at sandwich-type hemin/GQ DNAzyme electrochemical biosensor [138], with thiolated TLS11a aptamers attached to the gold nanoparticles modified GC electrode surface. The hemin-GQs were immobilized on Au-Pd core-shell nanoparticle-modified magnetic Fe3O4/MnO2 beads (Fe3O4/MnO2/Au-Pd). The hemin/GQ DNAzymes catalyzed the oxidation of hydroquinone with H_2_O_2_, amplifying the electrochemical current and improving the detection sensitivity.

## 4. Conclusions

This review discussed the recent advances on the electrochemical characterization, design, and applications of G-quadruplex electrochemical biosensors in the evaluation of metal ions, G-quadruplex ligands, and other small organic molecules, proteins, and cells. The incubation time and cations concentration dependence in controlling the G-quadruplex folding, stability, and formation of complex quadruplex-based nanostructures at the surface of carbon electrodes were discussed.

The different G-quadruplex electrochemical biosensors design strategies: the detection, via redox labels, of the G-rich DNA probe folding into a G-quadruplex structure after binding the analyte, the use of G-quadruplex aptamers, as recognition elements to capture the analyte, and the use of hemin/G-quadruplex DNAzymes, for electrochemical current amplification, were revisited.

G-quadruplex aptamer and/or DNAzymes-based biosensing strategies hold great promise for future applications in various fields, ranging from medical diagnostics and treatment to environmental monitoring and food safety. When compared with immunoassay-based biosensors, aptamer and/or DNAzymes-based electrochemical biosensors are particularly promising for the detection of small molecular targets, since it is difficult to produce highly specific antibodies for small molecules.

Understanding the G-quadruplex folding and stability at the solid-liquid interface of the electrochemical transducers is one of the fundamental challenges of the G-quadruplex electrochemical biosensors development and applications. The electroanalytical characterization using combined electrochemical and surface characterization techniques, of the G-quadruplexes redox behaviour and adsorption process, is crucial and emerges as an important and necessary step for the development of new, more sensitive, G-quadruplex electrochemical biosensors. More powerful signal amplification strategies are also estimated to be developed, in order to overcome the small concentrations of the molecular targets in real medical and environment samples, which may limit the biosensor sensitivity.
